# Changes of Amide Proton Transfer Imaging in Multiple System Atrophy Parkinsonism Type

**DOI:** 10.3389/fnagi.2020.572421

**Published:** 2020-09-30

**Authors:** Shuhua Li, Piu Chan, Chunmei Li, Haibo Chen, Min Chen, Wen Su, Kai Li, Na Lu, Lu Yu, Defa Chu, Pu-Yeh Wu

**Affiliations:** ^1^Department of Neurobiology and Neurology, Xuanwu Hospital, Capital Medical University, Beijing, China; ^2^Department of Neurology, Beijing Hospital, National Center of Gerontology, Institute of Geriatric Medicine, Chinese Academy of Medical Sciences, Beijing, China; ^3^Department of Radiology, Beijing Hospital, National Center of Gerontology, Institute of Geriatric Medicine, Chinese Academy of Medical Sciences, Beijing, China; ^4^Graduate School of Peking Union Medical College, Beijing, China; ^5^Department of Statistics, Beijing Hospital, National Center of Gerontology, Institute of Geriatric Medicine, Chinese Academy of Medical Sciences, Beijing, China; ^6^GE Healthcare, MR Research China, Beijing, China

**Keywords:** APT imaging, magnetic resonance imaging, biomarker, multiple system atrophy, parkinsonism

## Abstract

Multiple system atrophy (MSA), an atypical parkinsonism of alpha-synucleinopathies, has no specific biomarker of diagnosis. According to different combinations of symptoms, MSA can be classified as parkinsonism-type MSA (MSA-P) and cerebellar-type MSA (MSA-C; Watanabe et al., 2018). Amide proton transfer (APT) imaging is by far the most studied chemical exchange saturation transfer imaging for its sensitivity to mobile protons and peptides in tissues. We hypothesize that APT imaging may be a feasible biomarker of MSA-P. Twenty MSA-P patients and 20 age-matched normal controls were enrolled in this study and underwent MR exams on a 3.0-T MR scanner. Magnetization transfer spectra at 3.5 ppm were acquired at two transverse slices of the head, including the midbrain and the basal ganglia. Mann–Whitney *U* test was used to compare the asymmetrical magnetization transfer ratio (MTR_asym_) difference between MSA-P patients and normal controls. The APT MTR_asym_ values of MSA patients in the red nucleus (RN) (SN; *P* = 0.000), substantia nigra (*P* = 0.000), thalamus (*P* = 0.000), and putamen (*P* = 0.013) were significantly higher than those in normal controls. There was a negative correlation between APT MTR_asym_ and the score of part III of the Unified Parkinson Disease Rating Scale (*R* = −0.338, *P* = 0.044) in the putamen, while there was a positive correlation between the APT MTR_asym_ and the rate of motor symptom progression (*R* = 0.406, *P* = 0.017) in the RN. These findings suggest that APT MTR_asym_ changes are found and may be of value in the diagnosis of MSA-P.

## Introduction

Multiple system atrophy (MSA) is an adult-onset, health-threatening, sporadic, and progressive neurodegenerative disorder (Watanabe et al., 2002, 2018; Fanciulli and Wenning, 2015). Clinical manifestations caused by neuronal degenerations in striatonigral, olivopontocerebellar, and autonomic nervous systems include progressive dopamine-resistant parkinsonism, cerebellar ataxia, autonomic failure, and pyramidal signs (Fanciulli and Wenning, 2015; Watanabe et al., 2018). According to different combinations of symptoms, MSA can be classified as parkinsonism-type MSA (MSA-P) and cerebellar-type MSA (MSA-C; Watanabe et al., 2018) and diagnosed as probable and possible MSA. The time from onset of symptoms to diagnosis of probable MSA ranges from 1 to 10 years, with a median time of 2 years (Watanabe et al., 2002). Only 57.4% of MSA patients have both motor and autonomic manifestations within 2 years of disease onset, indicating that more than 40% of patients had symptoms involving only one system and could hardly be diagnosed in the first 2 years of the disease (Watanabe et al., 2002). Therefore, there is a great clinical need for biomarkers that can help early diagnosis of MSA. Although magnetic resonance imaging (MRI) is widely used for helping the diagnosis and differential diagnosis of MSA, conventional MRI has a limited role, particularly at the early stage (Watanabe et al., 2018). Previous studies have reported normal findings in conventional MRI for approximately 60% of MSA-P patients within 2 years of disease onset (Watanabe et al., 2002), and hence, other MRI methods that enable better early diagnosis of MSA are desirable.

Chemical exchange saturation transfer (CEST) imaging is a new method that has emerged in recent years (Henkelman et al., 2001; Vinogradov et al., 2013; Zhou et al., 2019), which enables the detection of endogenous or exogenous metabolites by observing the transferred saturation effects between the metabolite and water (Kogan et al., 2013). Amide proton transfer (APT) imaging, also known as amide CEST, is by far the most studied branch of CEST imaging because of its feasibility and robustness at 3.0 T and its sensitivity to mobile protons and peptides in tissues (Zhou et al., 2003). Other branches of CEST, such as amine CEST or hydroxyl CEST, are more feasible under higher magnetic field strengths. Previous studies have shown that APT imaging at 3.0 T has potential clinical value in diagnosis of brain tumors (Zhou et al., 2011), breast tumors (Dula et al., 2013), cerebral ischemia disease (Tietze et al., 2014; Harston et al., 2015; Jones et al., 2018), and Parkinson’s disease (PD; Li et al., 2014, 2015, 2017).

Previous studies have found neuronal losses and accumulation of abnormal cytoplasmic proteins (such as α-synuclein), gliosis, and microgliosis in the striatonigral pathway, including the red nucleus (RN), substantia nigra (SN), globus pallidus, thalamus, caudate, and putamen, in MSA-P patients (Ahmed et al., 2012). These pathological changes may be the basis for the possibility of APT imaging in MSA-P patients. We hypothesize that these pathological changes may lead to the modified APT imaging effects, and hence, APT imaging may be a feasible biomarker of MSA-P. The purpose of this study is to evaluate the feasibility of APT imaging in the diagnosis of MSA-P. We selected MSA-P and not MSA-C at the same time in this study for the following reasons. First, as a common type of MSA, it is difficult to differentiate PD, dementia with Lewy bodies, and progressive supranuclear palsy from MSA-P in the early stage of the disease. Thus, there is a great clinical demand for early detection and differential diagnosis of MSA-P. Second, if MSA-C and MSA-P are selected at the same time, more slices (slices covered RN and SN, basal ganglia, pons, cerebellum, and inferior olive) of the APT image will be performed with longer scan time, which will make the patients intolerant.

We conducted subgroup studies to evaluate the feasibility of APT imaging in diagnosis of MSA-P in different subgroups. There were two parts in subgroup studies. Patients were classified into probable and possible MSA-P subgroups according to the second consensus statement on the diagnosis of MSA (Gilman et al., 2008) in part 1, and patients were classified into conventional MRI positive MSA-P subgroup and conventional MRI negative MSA-P subgroup according to the visual assessment to conventional MRI in part 2. We compared the difference in APT imaging between each subgroup of MSA-P and normal controls (NCs) and evaluated the feasibility of APT imaging in subgroups of MSA-P. Furthermore, the correlations were evaluated between APT imaging signal intensity and the course of disease, age, the score of part III of the Unified Parkinson Disease Rating Scale (UPDRS III), rate of motor symptom progression, and Hoehn–Yahr scale (H&Y) score.

## Materials and Methods

### Subjects and Clinical Assessments

Twenty consecutive MSA-P patients (10 males and 10 females, mean age 66.5 ± 6.8 years) and 20 NCs (eight males and 12 females; mean age 67.9 ± 9.3 years) were recruited for this study. MSA-P patients were diagnosed according to the second consensus statement on the diagnosis of MSA (Gilman et al., 2008) by two experienced movement disorder specialists (19 and 24 years of experience).

The inclusion criteria for MSA-P patients were as follows: (Watanabe et al., 2018) probable MSA-P patients should have a sporadic, progressive adult-onset disorder including rigorously defined autonomic failure and poorly levodopa-responsive parkinsonism; and (Fanciulli and Wenning, 2015) possible MSA-P patients should have a sporadic, progressive adult-onset disease parkinsonism and at least one feature suggesting autonomic dysfunction plus another feature such as a clinical or neuroimaging abnormality (Gilman et al., 2008). Age-matched controls without neurological disorders were also enrolled. The exclusion criteria for all participants were as follows: (Watanabe et al., 2018) a history of exposure to antidopaminergic drugs; (Fanciulli and Wenning, 2015) head trauma; (Watanabe et al., 2002) stroke; (Vinogradov et al., 2013) central nervous system infection; (Henkelman et al., 2001) other neurologic or psychiatric diseases; and (Zhou et al., 2019) a structural lesion or hydrocephalus on brain MR images.

General information, medical history, and treatment information were collected and recorded *via* face-to-face consultation at the time of enrollment. The UPDRS III and H&Y scale were used to evaluate the motor impairment of MSA-P patients. The rate of motor symptom progression is defined as the UPDRS III score divided by the disease duration.

The protocol was approved by the ethics committee of Beijing Hospital (registration number: 2018BJYYEC-050–01). Each subject in this study signed informed consent forms prior to participation.

### MRI Acquisition

All participants underwent MRI examinations on a 3.0-T whole-body MRI system (SIGNA Pioneer, GE Healthcare, Milwaukee, WI, USA) equipped with a 16-channel head and neck coil. Conventional MRI, including axial T1-weighted imaging (T1WI), axial T2-weighted imaging (T2WI) with fat suppression, and diffusion-weighted imaging (DWI), was acquired to evaluate changes within and/or around the putamen and the brain stem and to exclude subjects with structural abnormality.

For each participant, the APT image was prescribed and performed on two slices with reference to T2WI images; one slice covered the RN and SN, and the other slice covered the basal ganglia region. For saturation pulse in APT, a train of four Fermi-filtered pulses with a duration of 400 ms and amplitude of 2 μT was used. For acquisition of APT, 2D echo-planar imaging (EPI) with the following parameters was used: TR/TE = 2, 500/26.2 ms, flip angle = 20°, field of view = 240 × 240 mm, matrix = 128 × 128, and slice thickness = 5 mm, and NEX = 2. The frequency offsets acquired were 5,000, 1,996, 768, 640, 576, 512, 480, 448, 416, 384, 320, 256, 192, 128, 96, 64, 32, 0, −32, −64, −96, −128, −192, −256, −320, −384, −416, −448, −480, −512, −576, −640, and −768 Hz.

### Data Processing

Hyperintense putaminal rim (HPR), putaminal atrophy, and hot cross bun were visually assessed by two radiologists (NL and LY, 2 and 6 years of experience, respectively) and two neurologists (SL and KL, 20 and 12 years of experience, respectively). The T2-hyperintense rim, located predominantly at the dorsolateral margin of the putamen, was evaluated as HPR in our study.

Magnetization transfer ratio asymmetry (MTR_asym_) was further performed on an AW4.6 GE workstation. The APT MTR_asym_ was calculated as MTR_asym_ (3.5 ppm) = Ssat (−3.5 ppm)/S0 − Ssat (+3.5 ppm)/S0, in which Ssat is the signal intensity with selective saturation, while S0 is not (Zhou et al., 2013). B0 inhomogeneity was corrected on a pixel-by-pixel basis. The data of APT MTR_asym_ were presented in percentage (%).

**Figure 1:**
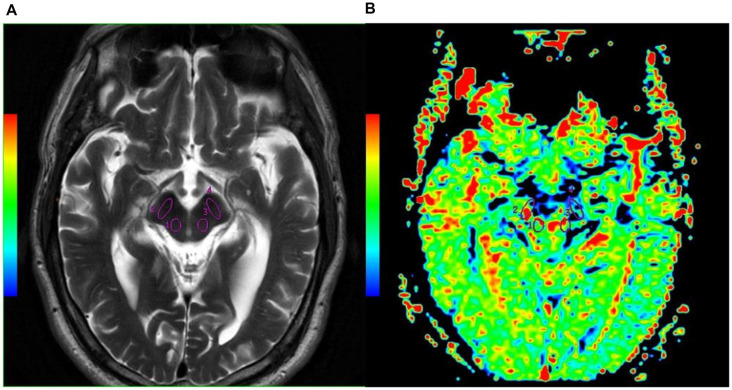
Examples of the definition of the regions of interest (ROIs) in the midbrain for quantitative analysis. **(A)** Axial T2WI and **(B)** axial image of amide proton transfer (APT). Refer to the T2WI to delineate the red nucleus (RN; Watanabe et al., 2002, 2018) and substantia nigra (SN; Vinogradov et al., 2013; Fanciulli and Wenning, 2015).

**Figure 2:**
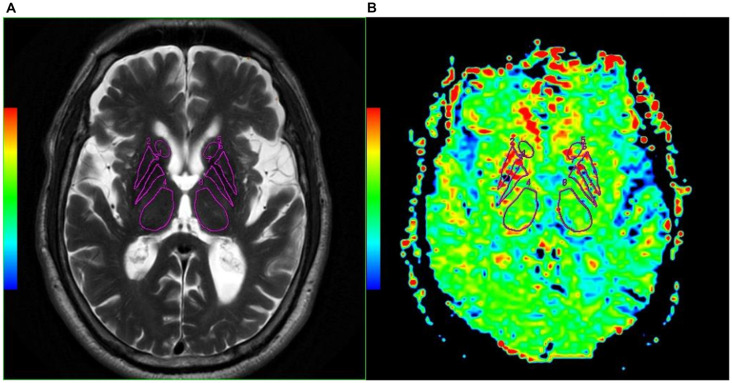
Examples of the definition of the ROIs in the basal ganglia for quantitative analysis. **(A)** Axial T2WI and **(B)** axial image of APT. Refer to the T2WI to delineate the caudate (Henkelman et al., 2001; Watanabe et al., 2018), putamen (Fanciulli and Wenning, 2015; Zhou et al., 2019), globus pallidus (Watanabe et al., 2002; Kogan et al., 2013), and thalamus (Zhou et al., 2003; Vinogradov et al., 2013).

T2WI was used to evaluate the function of APT images in our study mainly because T2WI could show SN and RN better than T1WI. Two radiologists (NL and LY) analyzed the quantitative MTR_asym_ images and compared the difference between MSA-P patients and NCs. Six regions of interest (ROIs), including RN, SN, globus pallidus, thalamus, caudate, and putamen of both hemispheres, were manually drawn based on T2WI images. ROIs from one representative subject overlaid on top of his own T2WI images and APT MTR_asym_ maps are as shown in Figures 1, 2.

### Statistical Analysis

All statistical analyses were performed using SPSS 25.0 (IBM, Armonk, NY, USA). The average APT MTR_asym_ at 3.5 ppm and the standard deviation were measured and calculated for each ROI. To compare the difference of APT MTR_asym_ (3.5 ppm) between MSA-P patients and NCs, a *t*-test was used if the data were normally distributed, and Mann–Whitney *U* test was used if the data were not normally distributed. A chi-square test was conducted to examine the clinical differences of categorical variables. Spearman rank correlation test was used to compare the correlation between APT MTR_asym_ and its related factors. The level of significance was set at *P* < 0.05.

## Results

### Demographic and Clinical Features

All MSA-P subjects had autonomic nervous system impairment and parkinsonism. Twelve MSA-P patients (seven females and five males) were diagnosed as clinically probable MSA-P patients, and eight MSA-P patients (two females and six males) as clinically possible MSA-P. Three probable MSA-P subjects had minor cerebellar symptoms, autonomic nervous system impairment, and parkinsonism. Two patients in possible MSA-P group had cerebellar symptoms. There was no statistically significant difference in age (*P* = 0.605) and gender (*P* = 0.525) between MSA-P and NCs. No statistically significant difference in age and gender was observed between each diagnostic category subgroup of MSA-P and NCs. The detailed information is shown in Table 1.

**Table 1:** Demographic information of the subjects.

	NCs	MSA-P	MSA-P	MSA-P	MSA-P	MSA-P	*z*/*χ*^2^	*P*-value
		probable	possible	MRI+	MRI−	NCs vs.		
Sample size	20	20	12	8	7	13		-
Age (years)^a^ mean ± SD	67.9 ± 9.3	66.5 ± 6.8	67.4 ± 7.4	68.5 ± 3.62	66.1 ± 4.1	68.7 ± 7.3	MSA-P, 0.522	0.605
							MSA-P probable, −0.234	0.815
							MSA-P possible, −0.055	0.956
							MSA-P MRI+, −0.166	0.868
							MSA-P MRI−, −0.390	0.679
Gender^b^ (female/male)	12/8	10/10	7/5	2/6	4/3	6/7	MSA-P, 0.404	0.525
							MSA-P probable, 0.009	0.926
							MSA-P possible, 2.800	0.094
							MSA-P MRI+, −0.018	0.895
							MSA-P MRI−, −0.179	0.493

*NCs, normal controls; MSA-P, multiple system atrophy—parkinsonism type; MSA-P MRI+, multiple system atrophy—parkinsonism type conventional MRI positive; MSA-P MRI−, multiple system atrophy—parkinsonism type conventional MRI negative; ^a^Independent *t*-test; ^b^Chi-square tests*.

Compared to possible MSA-P patients, the probable MSA-P patients showed higher average UPDRS III scores (*P* = 0.001). There was no statistically significant difference in disease duration (*P* = 0.516), H&Y scale (*P* = 0.208), and rate of motor symptom progression (*P* = 0.735) between probable and possible MSA-P patients. The detailed information is shown in Table 2.

**Table 2:** Basic clinical information of the MSA-P patients.

	MSA-P	MSA-P	MSA-P	*z*	*P*-value	MSA-P	MSA-P	*z*	*P*-value
		probable	possible			MRI+	MRI−		
Sample size	20	12	8	-	-	7	13	-	-
Disease duration (years), mean ± SD	2.5 ± 1.7	2.4 ± 1.8	2.5 ± 0.8	0.649	0.516	1.9 ± 1.2	2.9 ± 1.5	−1.427	0.154
Hoehn and Yahr staging, mean ± SD	2.9 ± 0.8	3.1 ± 2.6	2.6 ± 0.5	1.258	0.208	3.0 ± 0.8	2.8 ± 0.8	−0.449	0.653
UPDRS III, mean ± SD	31.5 ± 16.2	36.8 ± 17.1	23.9 ± 9.3	−3.390	0.001	36.1 ± 14.1	30.3 ± 17.3	−0.847	0.397
RMSP, mean ± SD	18.4 ± 16.0	18.9 ± 16.1	18.1 ± 12.0	0.338	0.735	26.9 ± 16.9	13.8 ± 10.7	−1.987	0.047

*MSA-P, multiple system atrophy—parkinsonism type; UPDRS III, the third part of the Unified Parkinson Disease Rating Scale; RMSP, rate of motor symptom progression. Mann–Whitney *U* test was used for the data listed in this table*.

### Conventional MRI Features for MSA-P Patients and NCs

No morphological abnormity was found within and around the putamen and the pons in NCs using conventional MRI. Hot cross bun was not found in our MSA-P patients, and putaminal changes were found in seven MSA-P patients (HPR in one patient, putaminal atrophy in three patients, and possible putaminal atrophy in three patients). So common abnormalities within and around the putamen (HPR and putaminal atrophy) can be observed in 35% of the MSA-P patients (7/20) at 3.0-T conventional MRI. The incidence of putaminal atrophy is 30%.

There were seven conventional MRI positive MSA-P patients (three males and four females) and 13 conventional MRI negative MSA-P patients (seven males and six females) in this study. No statistically significant difference in age and gender was observed between each subgroup (conventional MRI positive MSA-P subgroup and conventional MRI negative MSA-P subgroup) of MSA-P and NCs. Detailed information is shown in Table 1.

Compared with conventional MRI negative MSA-P patients, conventional MRI positive MSA-P patients showed higher rate of motor symptom progression scores (*P* = 0.047). No statistically significant difference in disease duration (*P* = 0.154), UPDRS scores (*P* = 0.397), and H&Y scale (*P* = 0.653) was observed between conventional MRI negative MSA-P patients and conventional MRI positive MSA-P patients. Detailed information is shown in Table 2.

### Changes of APT Imaging in MSA-P Patients

#### MSA-P vs. NCs

Compared with NCs, the MSA-P patients showed higher APT MTR_asym_ values in the regions of the RN (*P* = 0.000), SN (*P* = 0.000), thalamus (*P* = 0.000), and putamen (*P* = 0.001). In the regions of the caudate, the APT MTR_asym_ was higher in MSA-P patients than in NCs but did not reach statistical significance (*P* = 0.061). There was no significant difference in the APT MTR_asym_ values of the globus pallidus (*P* = 0.692) between MSA-P patients and NCs.

**Figure 3:**
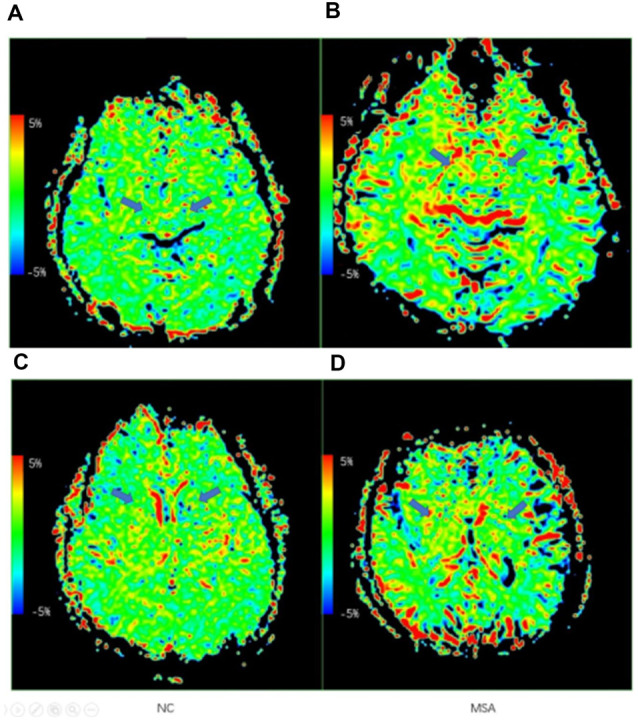
Examples of the APT maps of a normal control and an multiple system atrophy (MSA) patient. **(A,C)** Axial images of APT imaging in a normal control. **(B,D)** Axial images of APT imaging in an MSA patient. The arrows show the difference of the RN, SN, and basal ganglia region.

**Figure 4:**
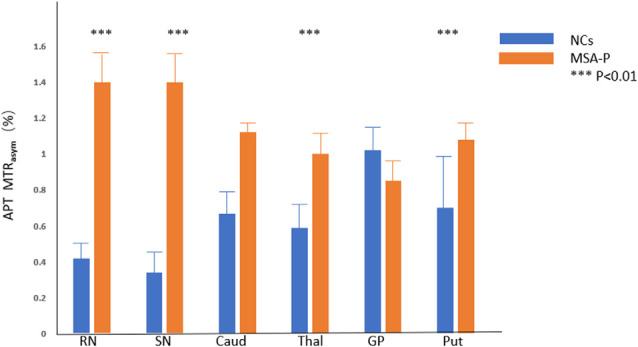
Comparison of average MTR_asym_ (3.5 ppm, %) between MSA patients and NCs. RN = red nucleus, SN = substantia nigra, Caud = caudate, Thal = thalamus, GP = globus pallidus, Put = putamen. ***p < 0.01.

Figure 3 shows the representative APT MTR_asym_ images of a probable MSA-P patient (female, 73 years old, H&Y scale of 3, disease duration of 3 years) and a NC (male, 60 years old). Table 3 and Figure 4 show the comparison of APT MTR_asym_ in the six ROIs between the MSA-P group and NCs.

**Table 3:** Changes in APT MTR_asym_ among MSA-P and controls.

APT MTR_asym_	NCs	MSA-P	*z*	*P*-value
(mean ± SE)	(*n* = 20)	(*n* = 20)		
RN	0.42 ± 0.08	1.4 ± 0.15	−4.838	0.000
SN	0.34 ± 0.14	1.40 ± 0.15	−5.682	0.000
Caud	0.67 ± 0.11	1.12 ± 0.16	−1.874	0.061
Thal	0.59 ± 0.11	1.00 ± 0.10	−4.291	0.000
GP	1.02 ± 0.09	0.85 ± 0.08	−0.396	0.692
Put	0.70 ± 0.08	1.08 ± 0.10	−3.278	0.001

*MSA-P, multiple system atrophy—parkinsonism type; RN, red nucleus; SN, substantia nigra; Caud, caudate; Thal, thalamus; GP, globus pallidus; Put, putamen. Mann–Whitney *U* test was used for the data listed in this table*.

#### Probable MSA-P and Possible MSA-P vs. NCs

Compared with NCs, probable MSA-P patients showed higher APT MTR_asym_ values in the RN (*P* = 0.000), SN (*P* = 0.000), thalamus (*P* = 0.001), caudate (*P* = 0.023), and putamen (*P* = 0.001). No statistically significant difference was found between probable MSA-P patients and NCs for the APT MTR_asym_ values in the globus pallidus (*P* = 0.836). Compared with NCs, possible MSA-P patients showed higher APT MTR_asym_ values in the RN (*P* = 0.006), SN (*P* = 0.000), and thalamus (*P* = 0.005). No statistically significant difference was found between possible MSA-P patients and NCs in the putamen (*P* = 0.050), globus pallidus (*P* = 0.650), and caudate (*P* = 0.164).

Compared with possible MSA-P patients, probable MSA-P patients showed higher APT MTR_asym_ values in the RN (*P* = 0.001), caudate (*P* = 0.013), and putamen (*P* = 0.010). No statistically significant difference was found between probable MSA-P and possible MSA-P patients in the SN (*P* = 0.516), thalamus (*P* = 0.868), and globus pallidus (*P* = 0.340). Detailed information is shown in Table 4.

**Table 4:** Changes in APT MTR_asym_ among probable MSA-P, possible MSA-P, and controls.

APT MTR_asym_ (mean ± SD)	NCs (*n* = 20)	MSA-P probable (*n* = 12)	MSA-P possible (*n* = 8)	*z*	*P*-value
RN	0.42 ± 1.03	1.56 ± 0.47	1.13 ± 1.55	NCs vs. MSA-P probable, −4.693	0.000
				NCs vs. MSA-P possible, −2.759	0.006
				MSA-P probable vs. possible, −3.390	0.001
SN	0.34 ± 1.02	1.31 ± 0.96	1.55 ± 0.50	NCs vs. MSA-P probable, −4.650	0.000
				NCs vs. MSA-P possible, −4.527	0.000
				MSA-P probable vs. possible, −0.649	0.516
Caud	0.67 ± 1.04	1.19 ± 0.82	1.08 ± 0.39	NCs vs. MSA-P probable, −2.274	0.023
				NCs vs. MSA-P possible, −1.392	0.164
				MSA-P probable vs. possible, −2.482	0.013
Thal	0.59 ± 0.63	0.85 ± 0.85	1.04 ± 0.33	NCs vs. MSA-P probable, −3.426	0.001
				NCs vs. MSA-P possible, −2.794	0.005
				MSA-P probable vs. possible, −0.167	0.868
GP	1.02 ± 0.38	0.85 ± 0.66	0.85 ± 0.33	NCs vs. MSA-P probable, −0.208	0.836
				NCs vs. MSA-P possible, −0.454	0.650
				MSA-P probable vs. possible, −0.953	0.340
Put	0.70 ± 0.64	1.10 ± 0.56	1.04 ± 0.32	NCs vs. MSA-P probable, −3.187	0.001
				NCs vs. MSA-P possible, −1.960	0.050
				MSA-P probable vs. possible, −2.573	0.010

*MSA-P, multiple system atrophy—parkinsonism type; RN, red nucleus; SN, substantia nigra; Caud, caudate; Thal, thalamus; GP, globus pallidus; Put, putamen. Mann–Whitney *U* test was used for the data listed in this table*.

#### Conventional MRI Positive MSA-P Patients and Conventional MRI Negative MSA-P Patients vs. NCs

Compared with NCs, conventional MRI positive MSA-P patients showed higher APT MTR_asym_ values in the RN (*P* = 0.001), SN (*P* = 0.000), thalamus (*P* = 0.012), and caudate (*P* = 0.045). No statistically significant difference was found between conventional MRI positive MSA-P patients and NCs in the putamen (*P* = 0.228) and globus pallidus (*P* = 0.515). Compared with NCs, conventional MRI negative MSA-P patients showed higher APT MTR_asym_ values in the RN (*P* = 0.000), SN (*P* = 0.000), thalamus (*P* = 0.000), and putamen (*P* = 0.000). No statistically significant difference was found between conventional MRI negative MSA-P patients and NCs in the caudate (*P* = 0.065) and globus pallidus (*P* = 0.325). No statistically significant difference was found between conventional MRI positive MSA-P patients and conventional MRI negative MSA-P patients in the above-mentioned regions. Detailed information is shown in Table 5.

**Table 5:** Changes in APT MTR_asym_ among MSA-P MRI+, MSA-P MRI−, and controls.

APT MTR_asym_ (mean ± SD)	NCs (*n* = 20)	MSA-P MRI+ (*n* = 7)	MSA-P MRI− (*n* = 13)	*Z*	*P*-value
RN	0.42 ± 1.03	1.46 ± 0.63	1.40 ± 0.29	NCs vs. MSA MRI+, −3.247	0.001
				NCs vs. MSA MRI−, −4.363	0.000
				MSA MRI+ vs. MSA MRI−, −1.064	0.287
SN	0.34 ± 1.02	1.59 ± 0.53	1.30 ± 0.93	NCs vs. MSA MRI+, −4.398	0.000
				NCs vs. MSA MRI−, −4.747	0.000
				MSA MRI+ vs. MSA MRI−, −0.330	0.741
Caud	0.67 ± 1.04	1.25 ± 0.23	1.08 ± 0.85	NCs vs. MSA MRI+, −2.004	0.045
				NCs vs. MSA MRI−, −1.844	0.065
				MSA MRI+ vs. MSA MRI−, −0.348	0.728
Thal	0.59 ± 0.63	0.84 ± 0.83	0.98 ± 0.58	NCs vs. MSA MRI+, −2.508	0.012
				NCs vs. MSA MRI−, −3.627	0.000
				MSA MRI+ vs. MSA MRI−, −0.121	0.904
GP	1.02 ± 0.38	1.02 ± 0.27	0.75 ± 0.65	NCs vs. MSA MRI+, −0.651	0.515
				NCs vs. MSA MRI−, −0.985	0.325
				MSA MRI+ vs. MSA MRI−, −1.211	0.226
Put	0.70 ± 0.64	0.99 ± 0.31	1.13 ± 0.56	NCs vs. MSA MRI+, −1.204	0.228
				NCs vs. MSA MRI−, −3.548	0.000
				MSA MRI+ vs. MSA MRI−, −0.938	0.348

*MSA-P MRI+, multiple system atrophy—parkinsonism type conventional MRI positive; MSA-P MRI−, multiple system atrophy—parkinsonism type conventional MRI negative; RN, red nucleus; SN, substantia nigra; Caud, caudate; Thal, thalamus; GP, globus pallidus; Put, putamen. Mann–Whitney *U* test was used for the data listed in this table*.

#### Summary for Changes of APT Imaging in MSA-P Patients

To summarize the main results (Watanabe et al., 2018), the APT MTR_asym_ values in the RN, SN, and thalamus were capable of differentiating MSA-P from NCs regardless of diagnostic categories and conventional MRI results; (Fanciulli and Wenning, 2015) the APT MTR_asym_ values in the putamen were also capable of differentiating MSA-P from NCs, especially for probable MSA-P patients; (Watanabe et al., 2002) the APT MTR_asym_ values were increased in the RN, SN, thalamus, and putamen in conventional MRI negative MSA-P patients, which indicates that APT imaging may be more sensitive than conventional MRI.

### Correlation Analysis

The APT MTR_asym_ at the putamen was negatively correlated with the UPDRS III score (*P* = 0.044), and the APT MTR_asym_ at the RN was positively correlated with rate of motor symptom progression (*P* = 0.017). There was no correlation between the course of disease, age, H&Y, and APT MTR_asym_. Detailed information is shown in Table 6.

**Table 6:** Correlation analysis of APT-MTR_asym_ in MSA-P patients.

	Age		Disease duration		H&Y		UPDRS III		Rate of progress	
	*R*	*P*	*R*	*P*	*R*	*P*	*R*	*P*	*R*	*P*
RN	−0.025	0.890	−0.135	0.446	0.388	0.124	0.154	0.386	0.406	0.017*
SN	−0.015	0.933	−0.281	0.101	0.201	0.440	0.058	0.746	0.163	0.356
Caud	−0.290	0.096	−0.012	0.942	0.332	0.164	0.044	0.797	0.009	0.960
Thal	−0.033	0.846	−0.071	0.670	0.020	0.934	−0.106	0.537	−0.014	0.934
GP	−0.248	0.133	−0.151	0.537	−0.298	0.215	−0.012	0.962	0.061	0.723
Put	0.320	0.065	−0.214	0.196	−0.345	0.148	−0.338	0.044*	−0.007	0.967

RN, red nucleus; SN, substantia nigra; Caud, caudate; Thal, thalamus; GP, globus pallidus; Put, putamen. Spearman rank correlation test was used for the data listed in this table. *P < 0.05.

## Discussion

This preliminary study demonstrated that the APT MTR_asym_ values in the RN, SN, thalamus, and putamen were higher in MSA-P patients as compared to those in healthy controls. A potential link with the pathophysiological mechanism of MSA-P was supported by the negative correlation between the APT MTR_asym_ at the putamen with the UPDRS III score.

MSA is an orphan disease (orphan number, ORPHAN102^1^; Fanciulli and Wenning, 2015), and the diagnosis of MSA at an early stage is challenging. A recent study reported that the clinicopathological consistency of MSA was only 62% (Koga et al., 2015). Multimodal MRI technology is often applied in the diagnosis of MSA patients (Pradhan and Tandon, 2017; Krismer et al., 2019). Several MRI findings have been reported in MSA-P patients, including the presence of a bilateral T2-hyperintense rim bordering the dorsolateral margins of the putamen (the “HPR sign”), T2-putaminal hypointensity (T2H), and atrophy of the putamen, middle cerebellar peduncles (MCP), cerebellum, or pons (Massey et al., 2012). In our study, the incidence of these morphological abnormalities within and around the putamen was only 35% in MSA-P patients at 3.0-T conventional MRI, and the incidence of putaminal atrophy was 30%. The incidence of these abnormalities was relatively low in MSA-P patients at conventional MRI, probably because our patients had a shorter duration of illness and relatively mild symptoms. Previous studies found that putaminal atrophy often occurs in advanced MSA-P patients, with a high specificity (92.3%) in distinguishing MSA-P from PD but a low sensitivity (44.4%; Feng et al., 2015), and our results were consistent with theirs. HPR incidence of our study was only 5%. The low incidence of HPR indicated that HPR was not a sensitive biomarker for early diagnosis of MSA-P. T2 “putaminal rim sign” and putaminal hypointensity were compared in one study using a 3.0-T MRI scanner; the result also indicated that HPR could not provide adequate diagnostic assistance in MSA-P patients (Lee et al., 2005). So far, the overall sensitivity of conventional MRI in diagnosing MSA-P especially at an early stage remains low.

In this study, the feasibility of using APT imaging in the diagnosis of MSA-P was evaluated. APT imaging has been reported to detect variations of proteins in diseases including brain tumors and PD. The accumulation of abnormal proteins in the central nervous system in a number of the neurodegenerative diseases has been reported in several pathological studies (Ito et al., 2009; Peden and Ironside, 2012; Fanciulli and Wenning, 2015). In MSA, oligodendroglia cytoplasmic inclusions (also known as Papp–Lantos bodies) are pathological hallmarks. Accumulated misfolded α-synuclein is a major component of the Papp–Lantos bodies (Spillantini et al., 1998; Ito et al., 2009; Peden and Ironside, 2012). These pathological characteristics form the basis of novel potential biomarker for MSA-P.

Our results showed that the APT MTR_asym_ values in the RN, SN, thalamus, and putamen were higher in MSA-P patients than in NCs. These observations may be attributed to the pathophysiological mechanism of MSA-P. In MSA-P, the primary pathological impairment regions are the nigrostriatal pathway, which may lead to abnormalities in the SN, RN, thalamus, and putamen (Wenning et al., 1997; Ahmed et al., 2012). The pathological characteristics were consistent with the results of this study that the APT MTR_asym_ difference was mainly observed in the RN, SN, thalamus, and putamen as the primary foci in MSA-P patients.

The main pathological abnormalities of MSA-P are neuronal loss and/or gliosis in the putamen and SN. One study found that MSA patients had more microglia in the brain than healthy controls (Salvesen et al., 2015). There were extensive microgliosis in the RN, putamen, globus pallidus, and subthalamic nucleus. The pathological hallmark of MSA is glial cytoplasmic inclusions (GCIs), and the key neuropathological manifestation of MSA is a broad spectrum of neuronal loss, gliosis, and myelin pallor. Neuronal loss may lead to a decrease of the APT MTR_asym_ due to a reduction of the water-exchanging chemicals, while gliosis, microgliosis, and GCIs may have an opposite effect. For MSA patients at the early stage of the disease, neuronal loss is relatively mild and the pathological change is often overlooked without special staining for gliosis (Wenning et al., 1997). Our findings of higher APT MTR_asym_ in MSA-P patients than in NCs suggested that the accumulation of abnormal cytoplasmic protein, gliosis, and microgliosis is more severe than neuronal loss on account of most of the MSA-P patients in our group. These findings support that APT imaging may be a potential biomarker in the diagnosis of MSA-P.

The results showed that the APT MTR_asym_ values in the globus pallidus had no statistically significant difference between MSA-P patients and NCs. This also may be attributed to the pathophysiological mechanism of MSA-P. Compared with the SN and putamen, the pathological changes of the globus pallidus were relatively mild (Wenning et al., 1997). According to the neuropathological grading of striatonigral degeneration (SND/MSA-P), the neuropathological changes (atrophy, neuronal loss, gliosis, and GCIs) in the globus pallidus were relatively mild, especially in grades I and II (Jellinger et al., 2005). For MSA-P patients at the early stage of the disease in our study, the accumulation of abnormal cytoplasmic protein, gliosis, and microgliosis may be equivalent to neuronal loss in the globus pallidus, the APT MTR_asym_ values were relatively normal in the globus pallidus.

Our results of subgroup analysis showed that the APT MTR_asym_ values in the RN, SN, and thalamus were capable of differentiating MSA-P from NCs regardless of diagnostic categories and conventional MRI results. The results indicate that the RN, SN, and thalamus may be the main foci of APT imaging in MSA diagnosis. The APT MTR_asym_ values in the putamen were also capable of differentiating probable MSA-P and possible MSA-P from NCs.

The APT MTR_asym_ values in the putamen were sensitive enough to distinguish conventional MRI negative MSA-P from NCs but not sensitive enough to distinguish conventional MRI positive MSA-P from NCs. HPR and putaminal atrophy were the main MRI features in our conventional MRI positive MSA-P patients. Posterior putaminal atrophy is related with neuronal loss and gliosis in the posterior putamen, and HPR is related to degeneration in the external capsule and/or lateral margin of the putamen (Matsusue et al., 2008). Compared with conventional MRI negative MSA-P patients, the conventional MRI positive patients had more severe neuronal loss, and loss of neurons led to a decrease in APT MTR_asym_ values. So compared with conventional MRI negative MSA-P patients, the conventional MRI positive MSA-P patients had lower APT MTR_asym_. As a result, APT MTR_asym_ values in the putamen were not sensitive enough to distinguish conventional MRI positive MSA-P from NCs. This may dynamically reflect the course of the disease. The APT MTR_asym_ values were increased in the RN, SN, thalamus, and putamen in conventional MRI negative MSA-P patients, which indicates that APT imaging may be more sensitive than conventional MRI to some extent.

We also did correlation analysis. The correlation coefficient was not very high, but it was still statistically significant. Correlation analysis found that there was a negative correlation between the APT MTR_asym_ of the putamen and the score of UPDRS III. As mentioned above, the decrease of APT MTR_asym_ was related to the loss of neurons. The result indicated that neuronal loss in the putamen may be closely related to the motor impairment in MSA-P patients. Wenning et al. also found that akinesia and rigidity were significantly correlated with the cell loss in the putamen, which was in line with our findings (Wenning et al., 1997). Our findings support that APT imaging may be a potential biomarker to evaluate motor function and the pathophysiological mechanism of MSA-P.

Correlation analysis also found that the APT MTR_asym_ at the RN was positively correlated with the rate of motor symptom progression. Previous work had reported that there were extensive microgliosis in the RN, putamen, and globus pallidus with neuronal loss in the putamen and globus pallidus (Salvesen et al., 2015). So compared with the putamen and globus pallidum, the RN may be a region that reveals microgliosis. The increase of APT MTR_asym_ in the RN may reflect the degree of microgliosis in the brain, and this microglia-dependent inflammatory processes may be related to the rate of motor symptom progression in MSA-P pathogenesis. Our findings support that APT imaging might also be applied to predict motor symptom progress of MSA-P. The above speculations, which were based on the results of this study and previous clinicopathological studies, need to be further investigated by prospective studies.

Several studies have found that APT MTR_asym_ in the SN of PD patients is lower than that in NCs (Tambasco et al., 2003; Anik et al., 2007; Morgen et al., 2011; Li et al., 2014). These results were related to the pathological abnormalities in the SN, which could lead to the decrease of water-exchanging chemicals. Based on these results, a number of studies have used CEST imaging as an imaging biomarker in diagnosis of PD (Li et al., 2014, 2015). A study found significantly increased APT MTR_asym_ in the globus pallidus, putamen, and caudate in PD patients compared with health controls (Fanciulli and Wenning, 2015). There was no significant difference in APT MTR_asym_ of the RN between PD and healthy controls (Anik et al., 2007; Morgen et al., 2011). Our results differ from previous studies on PD, suggesting that APT imaging may be helpful in differentiating MSA-P from PD. Further studies are required to evaluate the feasibility of APT imaging in differential diagnosis between MSA-P patients and PD in the future.

There were several limitations in this study. First, the sample size of the MSA-P group or the subgroup was limited. Longitudinal and large sample size studies are needed in the future. Second, only two slices of APT imaging were obtained in this study, which may lead to an inconsistency in ROI definition. Simultaneous comparison of the pons and cerebellum was insufficient; 3D volumetric acquisitions may allow better capturing of the brain regions of interest in the future. Third, ROI-based measurements were performed and compared in this study on a pathological basis. A pixel-by-pixel analysis based on the standard brain template may allow for the investigation of other brain regions. Finally, other auxiliary methods which can be applied as the diagnosis criteria of the MSA-P, for example, FDG-PET, were not compared.

In conclusion, our study identified that APT imaging may have potential value in diagnosis of MSA-P patients as well as in predicting the motor symptom progression of MSA-P. More investigations are needed for further evaluation of APT imaging in differential diagnosis of atypical parkinsonism.

## Data Availability Statement

The raw data supporting the conclusions of this article will be made available by the authors, without undue reservation.

## Ethics Statement

The studies involving human participants were reviewed and approved by The ethics committee of Beijing Hospital. The patients/participants provided their written informed consent to participate in this study.

## Author Contributions

SL was involved in all study phases. CL and MC assisted with the study design, helped execute the MRI examination, and contributed to manuscript preparation. HC assisted with the study design, referred subjects, and contributed to manuscript preparation. WS and KL referred subjects. NL and LY helped execute MRI examination and analyzed the quantitative MTR_asym_. DC assisted with statistical analysis and interpretation and provided manuscript critique. P-YW helped review the manuscript and provided manuscript critique. PC assisted with the study design, provided clinical oversight during study, and provided manuscript critique. All authors contributed to the article and approved the submitted version.

## Conflict of Interest

P-YW was employed by company GE Healthcare. The remaining authors declare that the research was conducted in the absence of any commercial or financial relationships that could be construed as a potential conflict of interest.

## Footnotes

^1^www.orpha.net
